# Intracranial Involvement in Takayasu’s Arteritis

**DOI:** 10.3390/diagnostics11111997

**Published:** 2021-10-27

**Authors:** Andrea Johnson, Derek Emery, Alison Clifford

**Affiliations:** 1 Division of Rheumatology, University of Alberta, Edmonton, AB T6G 2R3, Canada; ajj2@ualberta.ca; 2 Department of Radiology and Diagnostic Imaging, University of Alberta, Edmonton, AB T6G 2R3, Canada; demery@ualberta.ca

**Keywords:** Takayasu’s arteritis, vasculitis, intracranial, stroke, imaging

## Abstract

Takayasu’s arteritis (TAK) is a large-vessel vasculitis that targets the aorta and its major branches. Although extracranial vascular involvement is uniformly present in this disease, the frequency of intracranial involvement in TAK has not been well studied. We retrospectively reviewed the clinical and imaging records of patients diagnosed with TAK at a single Canadian university medical centre to determine the prevalence of intracranial vascular involvement. Intracranial vascular and non-vascular findings were described, and a review of the literature was performed. Of 20 patients with TAK, 12 had vascular neuroimaging completed. Intracranial vascular lesions were identified in 4 patients (33.3% of those with imaging available, 20% of all patients). The frequency of intracranial vessel involvement in TAK may be more common than appreciated. Imaging of both the intra- and extra-cranial vessels should be considered in these young patients.

## 1. Introduction

Takayasu’s arteritis (TAK) is a large-vessel vasculitis of unknown etiology, which, by definition, affects younger persons, and results in aneurysms, stenoses, and occlusions of the aorta and its major branch vessels. With an estimated annual incidence of 2.6 per million in North America [1], the clinical presentation of TAK is heterogenous and insidious. Some patients are asymptomatic, whereas others present with severe manifestations of end-organ ischemia. Among the breadth of symptoms that patients may experience, neurologic manifestations such as headache, light-headedness, vision changes, transient ischemic attack and stroke are common, estimated to be present in between 42% and 80% of TAK cases [2,3,4,5,6]. Neurologic manifestations in TAK most often develop as a result of vascular injury to the extracranial carotid, vertebral, or subclavian arteries.

Intracranial vascular involvement in TAK is considered uncommon; however, it has not been well studied. Existing literature suggests that intracranial vascular disease may affect between 5% and 50% of TAK patients [5,6,7,8,9,10,11,12]. This wide range is likely attributed to the differences in study designs, imaging modalities utilized, and the limited availability of neuroimaging studies among asymptomatic patients.

In this study, we aimed to determine the prevalence of intracranial vascular lesions in TAK patients at a single Canadian university centre, describe the most frequently observed lesions, and review the existing literature regarding the frequency of intracranial vascular disease in TAK.

## 2. Materials and Methods

The clinical records of patients diagnosed with TAK by rheumatologists at the University of Alberta, Edmonton, Canada, and seen between 2012 and 2018 were retrospectively reviewed. The electronic medical record database was searched by diagnostic code; then, identified records underwent complete chart review. For study inclusion, patients were required to have a confirmed clinical diagnosis of TAK by their treating rheumatologist, an onset of disease prior to age 50 years, and vascular imaging findings consistent with large-vessel vasculitis. Imaging findings consistent with large-vessel vasculitis were defined as the presence of wall thickening, vessel wall enhancement, vascular stenoses, occlusions, or aneurysms affecting the aorta and/or its major branches. Vessel wall thickening was considered in-keeping with vasculitis when diffuse and concentric over long segments, in the absence of significant atherosclerosis. Vessel wall enhancement was identified on MRI on post-gadolinium T1-weighted fat-saturated images. Demographics, including age, gender, ethnicity, and comorbidities, and clinical data, including the presence of systemic, vascular, and neurologic symptoms (specifically, headache, vision change, light-headedness, syncope, seizure, stroke, or other) at time of diagnosis and at time of neuroimaging were recorded. Laboratory results, including erythrocyte sedimentation rate (ESR), c-reactive protein (CRP), and complete blood count (CBC) were recorded. The presence of 1990 ACR classification criteria for TAK [13] were recorded, as were the imaging modality used and results of any large-vessel- and neuroimaging studies performed. A single neuroradiologist reviewed the images of available vascular neuroimaging (computerized tomography angiography (CTA), magnetic resonance angiography (MRA), conventional angiography) and non-vascular neuroimaging (CT, MR, positron emission tomography/computerized tomography (PET/CT)) studies and recorded the presence and characteristics of intra-cranial or extra-cranial head and neck lesions. Intracranial vascular lesions were defined as stenoses, occlusions, or aneurysms of the arteries within the calvarium, as identified on either CTA, MRA, or conventional angiograms of the head and neck vessels. Treatment with steroids, anti-platelet agents, and steroid-sparing therapies (including methotrexate, azathioprine, mycophenolate mofetil, cyclosporine, cyclophosphamide, anti-TNF therapy, and tocilizumab) were recorded. Study data were collected and managed using REDCap [14] electronic data capture tools hosted and supported by the Women and Children’s Health Research Institute at the University of Alberta. Descriptive statistics were used to analyze the cohort, including mean, median, and range as appropriate. Features of TAK patients with or without intracranial artery involvement were compared using Student’s *t*-tests (for continuous variables) or chi-squared tests (for discrete variables.) Ethics approval for this study was obtained through the Health Research Ethics Board—Health Panel, University of Alberta, project number Pro00067451. In addition, a literature search of PubMed using the search terms “Takayasu” and “intracranial” was performed to identify other reports describing the frequency of intracranial vasculitis in TAK patients. Additional records were identified by searching the references of key papers. Publications describing fewer than two TAK patients were not included.

## 3. Results

The initial diagnostic code search identified 24 possible patients in the electronic database. Upon complete chart review, 4 were excluded due to the presence of alternate diagnosis, resulting in 20 included TAK patients in this study. All patients had a clinical diagnosis of TAK by their rheumatologist, and all had imaging evidence of large-vessel vasculitis involving the aorta and/or its major branches, as defined above. All patients were assessed by their rheumatologist more than once, with a median number of 9.5 clinical assessments (range 2–47). All patients met the 2012 Chapel Hill Consensus Conference nomenclature definition of Takayasu’s arteritis [15], and 12 of 20 also met the 1990 American College of Rheumatology (ACR) criteria for TAK [13]. Of the 20 TAK patients identified, 18 (90%) were female, with an average age at diagnosis of 29.5 years (range 13–48). 

Common comorbidities among TAK patients included hypertension in seven patients (35%), previous autoimmune disease in five (25%), and smoking history in six (30%). Among the previous autoimmune diseases, four patients (20%) had concomitant inflammatory bowel disease, one patient (5%) had psoriatic arthritis, one patient (5%) had uveitis, one patient (5%) had Hashimoto’s thyroiditis, and one patient (5%) had Grave’s disease. Some patients had several co-existing autoimmune conditions. The most common symptoms at diagnosis were limb claudication (six patients, 30%), and headache (six patients, 30%). Neurologic symptoms were present in ten (50%) TAK patients, of which eight patients (40%) had neuroimaging. Table 1 details complete baseline patient demographics and presenting symptoms. Symptoms were present for a median of 2 months prior to diagnosis (range 0 to 36 months). 

### 3.1. Management

Initial treatment included steroids in 18 (90%) of 20 TAK patients; 15 patients (75%) also received concomitant steroid-sparing agents, as outlined in Table 1. 

### 3.2. Extracranial Large-Vessel Imaging Results

**Figure 1:**
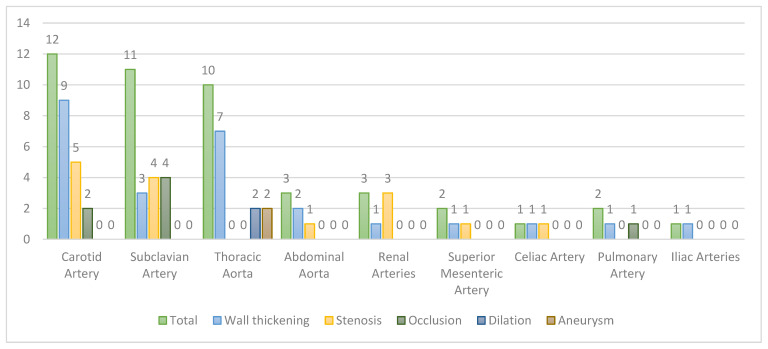
Extracranial vessel abnormalities found on imaging including wall thickening, stenosis, occlusion, dilation, and aneurysm.

All patients had extra-cranial large-vessel imaging available, with either CT angiography or MR angiography (CTA performed in 9/20 patients, MRA performed in 12/20 patients, 1 had both CTA and MRA, and 11/20 patients also underwent PET). Extracranial lesions typical of TAK were present in all 20 (100%), most commonly affecting the extracranial carotid arteries (12 patients, 60%), subclavian arteries (11 patients, 55%), and thoracic aorta (10 patients, 50%). The abdominal aorta was involved in three patients (15%), renal arteries in three (15%), superior mesenteric artery in two (10%), pulmonary arteries in two (9.5%), celiac artery in one (5%), and the iliac artery in one (5%). Vascular imaging abnormalities included wall thickening (in 11 patients, 55%), vascular stenoses (in 10 patients, 50%), occlusive lesions (in 6 patients, 30%), and aneurysms (in 5 patients, 25%). Details of extracranial imaging findings are available in Figure 1.

### 3.3. Neuroimaging Results

Neuroimaging was performed in 15 of 20 patients (75%); however, images were unavailable to review in 2 patients, and 1 patient had non-vascular neuroimaging only, leaving 12 TAK patients with vascular neuroimaging studies available for review. Eight patients had vascular neuroimaging completed at the time of diagnosis, although in three patients, the first study was delayed for more than 1 year after initial TAK diagnosis (median 0, range 0–24 months). At the time of vascular neuroimaging, 8 of 12 patients (66.7%) had neurologic symptoms, including stroke (4, 33.3%), vision changes (3, 25.0%), headache (2, 16.7%), or light-headedness (2, 16.7%). Intracranial vascular lesions were identified in four patients (33.3% of those with vascular neuroimaging available, 20% of all patients). Three patients (15%) had involvement of the middle cerebral arteries, two patients (10%) had involvement the intracranial portion of the internal carotid arteries, and a single patient (5%) had involvement of the anterior cerebral artery. The clinical and radiographic details of all 12 patients with vascular neuroimaging studies available are summarized in Table 2. 

### 3.4. Comparison of TAK Patients with and without Intracranial Vascular Disease

TAK patients with and without intracranial vascular disease were compared with respect to age at diagnosis, frequencies of smoking (current or former), hypertension, the presence of carotid artery involvement, c-reactive protein (as a surrogate for disease activity), and Numano classification (see Table 3). All patients with intracranial vascular lesions had involvement of the extracranial carotid arteries and great arch vessels (Numano classification I, IIA, or V). No statistically significant differences were observed. A detailed description of the four patients with intracranial vascular lesions is found below.

### 3.5. Case 1

**Figure 2:**
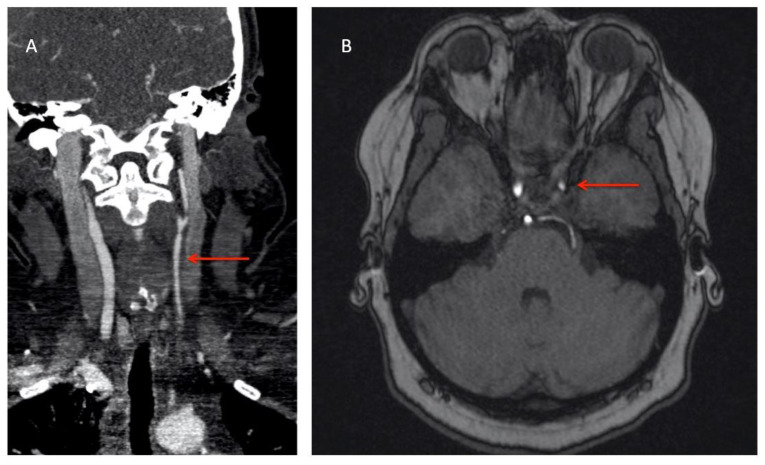
(**A**) CT-A demonstrating extensive, diffuse narrowing of the left common carotid artery and extracranial internal carotid artery; (**B**) MR brain with and without contrast showing left internal carotid artery terminus narrowing.

Patient 1 was a 30-year-old female who presented with new-onset hypertension, and after medical attention it was found that their left brachial pulse was absent. The patient also reported a several-year history of left arm claudication, occasional headaches, and scalp tenderness. Inflammatory markers were elevated, with ESR 28 mm/h, and CRP 21.3 mg/L. CT-A of the chest, abdomen, and pelvis revealed a long segment (7 cm) of left subclavian stenosis/occlusion, and diffuse stenosis to the left common carotid artery. A diagnosis of TAK was performed, and treatment with prednisone, methotrexate, folic acid, and ASA were initiated. Despite treatment, inflammatory markers remained persistently elevated (CRP 21 mg/L), left arm claudication worsened, and the patient reported new episodes of slowed thinking and light-headedness. A CT-A of the head and neck arteries, followed by the MR brain with and without enhancement, revealed high-grade stenosis of the left cavernous carotid artery (see Figure 2) with mild multi-segmental stenoses in the skull base portion of the left internal carotid artery, and irregularities of the bilateral MCAs and left ACA. Diffuse vessel wall enhancement was noted. Due to worsening symptoms, elevated inflammatory markers, and intracranial vessel involvement, prednisone was increased, and infliximab 5 mg/kg was added. The patient’s symptoms improved, serial imaging stabilized, and brain perfusion study was normal. Follow-up imaging one year later revealed stable lesions with the ongoing diffuse narrowing of the left internal carotid artery and circumferential enhancement, as well as ongoing stenoses of left M1 and left A1 branches, but no new vascular lesions.

### 3.6. Case 2

Patient 2 was a 48-year-old female who presented with dysarthria and left arm weakness, and was found to have a right MCA ischemic stroke. Past medical history was significant for hypertension, dyslipidemia, and previous smoking history. Inflammatory markers were normal at the time of diagnosis. CTA of the head and neck revealed long segments of circumferential thickening of bilateral common, internal, and external carotid arteries with lumenal irregularities. Significant narrowing of the right internal carotid artery extended up into the cavernous portion of internal carotid artery. A small right frontoparietal MCA embolic infarct was also noted. Thoracic aorta and subclavian arteries appeared normal on MRA; however, the abdominal aortic wall was thickened and enhanced with gadolinium contrast. A diagnosis of TAK was made by her treating rheumatologist. She was treated with prednisone taper, followed by mycofenolate mofetil upon diagnosis. Follow-up MRA 1 month later revealed improved wall enhancement, suggesting a response to treatment, and repeat imaging several years later revealed stability of these lesions.

### 3.7. Case 3

Patient 3 was a 29-year-old female who presented with acute onset sensory loss of the right face, arm, and leg, and was found to have left MCA ischemic stroke. The patient denied any preceding constitutional or vascular symptoms. She had a past medical history of psoriatic arthritis and ulcerative colitis. Inflammatory markers at presentation were elevated, with an ESR 58 mm/h and CRP of 12.7 mg/L. CTA and MRA of the head and neck vessels, followed by conventional angiogram of the brain, were performed. Imaging studies revealed wall thickening of the aortic arch and proximal great vessels, mild narrowing in both distal common carotid arteries, severe stenosis of the left subclavian artery, and moderate stenosis of the right subclavian artery. Intracranially, the left MCA was occluded. She was diagnosed with TAK by rheumatology, and treated with pulse steroids, followed by subcutaneous weekly methotrexate. Subsequent follow-up showed clinical stability and improvements in the radiographic appearance of the subclavian artery stenosis.

### 3.8. Case 4

Patient 4 was a 13-year-old female presenting with acute onset left hemiparesis, aphasia, and vision changes, on a background history of 2 months of headaches. On examination, no pulses were detectable in the left arm. Inflammatory markers were normal, but initial non-vascular imaging with CT revealed a right frontal intracerebral hematoma and left parietal infarcts. Multiple assays, including CT, MR, CT-A, MR-A, and conventional angiography, were performed. Severe luminal stenoses approaching 70% were noted in the left common carotid artery, left carotid bifurcation, right common carotid, and proximal right subclavian arteries. The left subclavian artery was completely occluded. Stenoses of the right MCA, affecting M1 and M2 branches, were also noted. TAK was diagnosed by a pediatric rheumatology. She was treated with prednisone, aspirin, and azathioprine. Symptoms improved, and lesions stabilized on follow-up imaging.

### 3.9. Review of Literature

A review of the literature identified eight additional publications describing the intracranial vasculature in two or more TAK patients [5,6,7,8,9,10,11,12]. Table 4 presents a summary of the reviewed papers. In addition to the present study, six retrospective series were included, as well as two prospective studies. Various imaging modalities were utilized, including transcranial Doppler, computerized tomography angiography (CTA), magnetic resonance angiography (MRA), single positron emission computed tomography (SPECT), and conventional angiography. Taken together with our data, the intracranial vessels were described in a total of 300 patients with TAK, and of those, 74 (14.9% of total or 24.7% of those with intracranial imaging) demonstrated intracranial vascular involvement [5,6,7,8,9,10,11,12]. The reported frequency of intracranial involvement in TAK ranged widely in individual studies, from 4.9% to 41.9% [5,6,7,8,9,10,11,12], as shown in Table 4. Among the prospectively conducted studies, intracranial lesions were identified in 1 of 4 (25%) [10], and 5 of 21 (24%) TAK patients with or without neurologic symptoms [9], respectively. Similarly, the largest study, a retrospective review of 135 hospitalized TAK patients in China, identified 32 patients, or 23.7%, with intracranial vascular involvement [12]. Although no differences in the frequency of headache, dizziness, loss of consciousness, or vision change were reported compared to those without intracranial vascular involvement, patients with intracranial disease were significantly more likely to have hypertension (46.9% vs. 26.2%, *p* < 0.05), coronary artery disease (21.9% vs. 6.8%, *p* < 0.05), and coronary artery involvement by TAK (43.8% vs. 14.6%, *p* < 0.01.) The intracranial segment of internal carotid artery was the most commonly involved intracranial vessel (affected in 19/32, 59.4%), followed by the middle cerebral arteries (12/32, 37.5%), anterior cerebral arteries (11/32, 34.4%), and posterior cerebral arteries (10/32, 31.3%). In addition, a recent retrospective study of 122 TAK patients from China, of whom 64 (52.5%) had intracranial imaging available, was published [16]. In this study, middle cerebral artery involvement was identified in 27 patients (22.1%), anterior cerebral artery involvement was seen in 8 (6.5%), and posterior cerebral artery involvement was seen in 6 patients (4.9%); however, because some patients may have had involvement of multiple cranial arteries, the total number of patients with intracranial vessel involvement was unclear; thus, it was not included in our compiled analysis. 

## 4. Discussion

Neurologic manifestations are very common in TAK [2,3,4,5,6]. A recent retrospective review of 610 TAK patients found that nearly half (44.9%) experienced symptoms such as dizziness, headache, vision changes, syncope, stroke, or transient ischemic attack (TIA) [2]. Serious cerebrovascular events such as stroke or TIA occur in between 9% and 20% of TAK patients over the course of their disease [2,4,17,18,19,20], and among TAK patients who suffer a stroke, 59% experience persistent neurologic impairment upon follow-up [18], emphasizing the potential for severe long-term impacts of this disease on future health.

Several mechanisms likely contribute to cerebrovascular events in TAK patients. Central nervous system ischemia most commonly occurs due to stenoses within the extra-cranial arteries of the neck, such as carotids or vertebral arteries [4,6,17,21]. In select cases, however, subclavian steal has been implicated [5,6,7], as has arterial thrombosis [19], and accelerated atherosclerosis [19,22,23]. Embolic phenomena or micro-emboli, secondary to proximal aortic lesions or cardiac valvular disease, have also been described [6,20,24,25,26], particularly in association with aortic regurgitation [6,21,27]. In some cases, the presence of concomitant hypercoagulable states [19], such as positive lupus anticoagulant [28] or Crohn’s disease [29], may contribute to cerebrovascular events. More rarely, dissection [30] or intracranial haemorrhage may occur [6,20]. Lastly, the presence of intracranial vasculitis, although generally thought to be rare in this disease, has been increasingly recognized in TAK patients [5,6,7,8,9,10,11,12] with neurologic symptoms. 

In this small series of 20 TAK patients, neurologic symptoms were present in 50% and intracranial vascular abnormalities suggestive of intracranial vasculitis were noted in 20% overall, or 33.3% of those with dedicated neurovascular imaging, suggesting that intracranial vascular involvement may be present in a significant minority of TAK patients. Of note, all four of our patients with intracranial vascular disease had neurologic symptoms, and stroke was present in three of four (75%) at presentation. Our findings are broadly in-keeping with the existing literature, which suggests that intracranial involvement in TAK may be identified in approximately 25% of patients in whom such imaging is performed. The frequency of intracranial involvement varies considerably in individual studies (from 4.9% to 41.9%), likely as a result of study design, reporting bias, patient selection, and the type of imaging modality performed. Significant variability in the clinical presentation of TAK has also been observed across different geographic regions [31] and ethnicities [32], suggesting another possible source of differences. In our cohort, we were unable to assess the role of ethnicity due to inconsistent documentation in medical records. 

The baseline demographics of this TAK patient cohort are consistent with those described in the literature [33]. Stroke or TIA were observed in 27.3% of our TAK patients, compared to 9–20% in recent reports [2,4,17,18,19,20]. Similar to other studies, in our patients, the intracranial arteries most likely to be affected were the middle cerebral arteries, followed by the intracranial portions of the internal carotid arteries [13,34]. In our patients, intracranial vascular lesions were identified at the time of first dedicated vascular neuroimaging, which was usually performed early in the disease course. Given small patient numbers, we were not able to identify any statistically significant risk factors for intracranial artery involvement; however, we note that all four (100%) of our TAK patients with intracranial vascular lesions also had involvement of the great arch vessels (Numano types I, IIa, and V), and more specifically, all four had involvement of the extracranial carotid arteries. This raises the hypothesis that TAK patients with extracranial carotid artery involvement may be more likely than those without to develop intracranial vascular disease; however, further study is needed. 

This study is limited by the retrospective nature and small sample size. The majority of patients who had complete neuroimaging had concurrent neurologic symptoms, potentially falsely increasing the prevalence of intracranial disease found. Additionally, in most of our patients, conventional angiograms were not performed; therefore, the involvement of smaller intracranial arteries could have been missed. 

Currently, many vasculitis experts advocate for serial large-vessel imaging of the aorta and major branch vessels in TAK patients, in order to detect new lesions indicative of ongoing disease activity that may be clinically silent by history or physical exam [16]. There is a lack of evidence regarding the value of routine imaging of the intracranial vessels in this disease, however. In the present study, intracranial vasculitic lesions were identified in one in three patients in whom such imaging was performed, and were typically present at the time of first neuroimaging, suggesting that intracranial involvement may be more common than previously suspected. Intracranial vascular lesions were only observed in those patients who had stenoses of the extra-cranial carotid arteries. Obtaining both intra- and extra-cranial vascular imaging in TAK patients at baseline may provide a more complete assessment of the extent of patients’ disease and provide an opportunity for monitoring disease progression. Given the potentially serious neurologic consequences, gaining an improved understanding of the true frequency of intracranial involvement in TAK will be important to help inform recommendations for disease monitoring in these young patients. Prospective studies are needed.

## Figures and Tables

**Table 1:** Baseline characteristics of TAK patients.

Patient Baseline Characteristics (20 Patients)	
Age (years)	29.5 (13–48)
Female	18 (90%)
Comorbidities	
Hypertension	7 (35%)
Smoker	
Ex-smoker	3 (15%)
Current smoker	2 (10%)
Other autoimmune diseases	5 (25%)
Inflammatory Bowel Disease	4 (20%)
Symptoms	
Limb claudication	6 (30%)
Headache	6 (30%)
Stroke	6 (30%)
Previous TIA	2 (10%)
Vision changes	5 (25%)
Chest Pain	5 (25%)
Arthralgia	3 (15%)
Fever	2 (10%)
Weight loss (>5 kg)	2 (10%)
Scalp tenderness	1 (5%)
Paresthesia	1 (5%)
Abdominal pain	1 (5%)
Physical findings	
Loss of pulse	8 (40%)
Arm	5 (25%)
Radial	3 (15%)
Brachial	1 (5%)
Leg	1 (5%)
Carotid	2 (10%)
Asymmetric blood pressure (>10 mmHg)	7 (35%)
Bruit	6 (30%)
Weakness	4 (20%)
Sensation loss	1 (5%)
Baseline bloodwork	
ESR (mm/h)	34 (1–196)
CRP (mg/L)	21.3 (0.7–108)
Treatment	
Steroids	18 (90%)
Steroid-sparing medication	15 (75%)
Methotrexate	8 (40%)
Azathioprine	3 (15%)
Mycophenolate mofetil	2 (10%)
Rituximab	1 (5%)
Cyclophosphamide	1 (5%)

Data are expressed as numbers (percent) and median (range). Abbreviations: TIA; transient ischemic attack; ESR, erythrocyte sedimentation rate; CRP, c-reactive protein.

**Table 2:** Summary of TAK patients with neuroimaging completed.

Patient	Age, Sex	Comorbidities	Description of EC Vascular Findings	EC Imaging Modality	Numano	Description of IC Vascular Findings	IC Non-Vascular Findings	IC Imaging Modality	Treatment
1	30 F	Grave’s disease IBD Uveitis	-Stenosis/occlusion of L subclavian artery -Stenosis of L common carotid artery	CTA, MRA	I	-Stenosis of L cavernous carotid artery -Stenoses of bilateral MCAs and L ACA with diffuse wall enhancement	None	CTA MRA MRI	MTX, GC, IFX
2	48 F	HTN Dyslipidemia Ex-smoker	-Stenoses and bilateral common carotid arteries -Stenosis R internal carotid artery -Abdominal aortic wall thickening and enhancement	CTA MRA CT PET	V	-Stenoses of R cavernous carotid artery	-R MCA infarct	CTA CT	MMF, GC
3	29 F	Psoriatic arthritis IBD	-Wall thickening of the aortic arch and proximal great vessels -Stenoses of both common carotid arteries -Stenoses of L and R subclavian arteries	MRA	IIa	-Occluded L MCA	-L MCA infarct	CTA MRI CT	MTX, GC
4	13 F	Enlarged adenoids Tympano- plasty	-Stenoses of bilateral common carotid arteries -Stenosis of R subclavian artery -Stenosis/occlusion of L subclavian artery	CT MRA PET	I	-Stenoses of R MCA, affecting M1 and M2 branches	-R frontal intracerebral hematoma	CT CTA MRA MRI Conventional angiogram	GC, AZA
5	19 F	Previously healthy	-Stenosis and wall enhancement of bilateral common carotid arteries -Occlusion of L subclavian artery -Stenosis of infrarenal abdominal aorta	CTA MRA PET	V	None	None	CTA MRA	GC, AZA
6	28 F	Smoker	-Long segment stenosis and wall thickening of L common carotid artery	CTA MRA	I	None	None	CTA MRA	MTX, GC
7	36 F	HTN IBD	-Wall thickening of the distal thoracic and abdominal aorta -Wall thickening of proximal celiac, SMA, and bilateral common iliac arteries	CT CTA MRA PET	III	None	None	MRA MRI	GC
8	35 F	Previously healthy	-Diffuse wall thickening of L common carotid artery	CTA MRA PET	I	None	None	CT CTA MRA	GC, AZA
9	32 F	Endometriosis Asthma	-Diffuse wall thickening of ascending aorta, and L subclavian artery -Fusiform dilation of descending thoracic aorta -Occlusion of R pleural artery	CT CTA MRA PET	IIb	None	-Bilateral frontal micro- angiopathy	CT MRI MRA	MMF
10	34 F	HTN Aortic insufficiency	-Stenosis of innominate artery -Stenoses and ectasia of bilateral common carotid arteries -Stenosis and enhancement of L subclavian artery	CTA MRA	I	None	None	CT MRI MRA Conventional angiogram	GC, MMF
11	45 M	Previously healthy	-Wall thickening of aortic arch and descending thoracic aorta -Wall thickening of innominate, L common carotid and L subclavian arteries -Attenuation of L common carotid and R subclavian arteries	MRA PET	IIb	None	None	CT CTA MRI	MTX, GC
12	33 F	HTN Hypothyroidism Anxiety	-Wall thickening and dilatation of aortic arch -Stenosis of L common carotid artery -Occlusion of R common carotid artery	CTA MRA PET	IIa	None	-Multiple posterior circulation ischemic lesions	CTA MRI	CYC, GC

Summary of twelve TAK patients in whom neuroimaging was completed. Abbreviations: TAK, Takayasu’s arteritis; EC, extracranial; IC, intracranial; IBD, inflammatory bowel disease; L, left; R, right; CTA, computerized tomography angiography; MRA, magnetic resonance angiography; PET, positron emission computed tomography; MTX, methotrexate; GC, glucocorticoids; AZA, azathioprine; MMF, mycofenolate mofetil; CYC, cyclophosphamide.

**Table 3:** Comparison of TAK patients with and without intracranial artery involvement.

	TAK Pts without IC Involvement (8)	TAK Pts with IC Involvement (4)
Age (years)	33.5 (19–45)	29.5 (13–48)
Smoking	1 (12.5%)	1 (25%)
HTN	2 (25%)	2 (50%)
CRP (mg/L)	13.6 (8.3–108)	12.7 (0.7–21.3)
Carotid Artery Involvement	6 (75%)	4 (100%)
Numano I	3 (37.5%)	2 (50%)
IIa	1 (12.5%)	1 (25%)
IIb	2 (25%)	0
III	1 (12.5%)	0
IV	1 (12.5%)	0
V	1 (12.5%)	1 (25%)

Results listed as median +/− range, and number (percentage). Abbreviations: IC, intracranial; HTN, hypertension; CRP, c-reactive protein. No statistically significant differences were identified with any variable.

**Table 4:** Intracranial vasculitis in the TAK literature review.

Author (Year)	Country	# TAK Patients in Study	# Pts with IC Vessel Evaluation	Imaging Modality	# Pts with IC Vessel Disease (%)	Study Type	IC Vessel Lesions Identified
Bond (2017)	USA	79	67	Doppler, CTA, MRA, MRI	9 (11.4%)	Retrospective	Stenosis, occlusion, aneurysm
Kim (2005)	Republic of Korea	70	27	Doppler, CTA, CT, MRA, MRI	6 (8.6%)	Retrospective	Stenosis, occlusion
Ringleb (2005)	Germany	17	17	Doppler, MRA	7 (41.2%)	Retrospective	Stenosis, occlusion
Cantu (2000)	Mexico	21	21	Doppler, MRA	5 (23.8%)	Prospective	Stenosis
Hoffmann (2000)	South Africa	142	10	Doppler, CTA, MRA, SPECT, Angiogram	7 (4.9%)	Retrospective	Stenosis, occlusion
Suwanwela (1998)	Thailand	4	4	Doppler, MRA, Angiogram	1 (25%)	Prospective	Stenosis, occlusion
Takano (1993)	Japan	7	7	Angiogram	3 (42.9%)	Retrospective	Stenosis, occlusion
Guo (2020)	China	135	Not specified	CTA, MRA	32 (23.7%)	Retrospective	Stenosis, occlusion
Johnson (2021)	Canada	20	12	CT, CTA, MRI, MRA, SPECT, Angiogram	4 (20%)	Retrospective	Stenosis, occlusion
Total	Various	495	300	Varied	74 (14.9%)	Varied	Varied

Abbreviations: TAK, Takayasu’s arteritis; #, number; IC, intracranial; Doppler, transcranial Doppler; CTA, computerized tomography angiography; MRA, magnetic resonance angiography; SPECT, single positron emission computed tomography; angiogram, conventional angiogram.

## Data Availability

The data presented in this study are available on request from the corresponding author.
